# A case of the blue blues: the safety issue of recognizing and avoiding the retinal toxicity of methylene blue compared to brilliant blue and trypan blue

**DOI:** 10.1186/s40942-025-00759-1

**Published:** 2025-12-05

**Authors:** Sidra Zafar, Jose S. Pulido, Samir N. Patel, Yoshihiro Yonekawa, Jordan D. Deaner

**Affiliations:** 1 Wills Eye Hospital Retina Service, Mid Atlantic Retina, Thomas Jefferson University, 840 Walnut Street, Suite 1020, Philadelphia, PA 19107 USA; 2 The Vickie and Jack Farber Vision Research Center, Philadelphia, PA USA

## Abstract

While blue vital dyes remain indispensable in ophthalmic surgery, medication errors, particularly the inadvertent use of methylene blue instead of trypan blue and brilliant blue, pose a significant risk to patient safety. Methylene blue has been reported to cause damage to anterior segment structures, We reported a case of inner retinal toxicity from injection of methylene blue into the vitreous cavity resulting in profound vision loss. Standardization, vigilant verification, and safer packaging are critical to preventing these never events.

In this issue, we described a case of retinal toxicity associated with the inadvertent injection of methylene blue into the vitreous cavity during vitrectomy surgery. The subsequent vision loss was severe and irreversible.

Blue vital dyes aid in the visualization of ocular tissue and are an integral part of ophthalmic surgery [1]. Trypan blue is commonly used by anterior segment surgeons while performing capsulorhexis during cataract surgery and is also used to stain Descemet’s membrane during DSAEK (Descemet’s stripping endothelial keratoplasty). Brilliant Blue is similarly used by vitreoretinal surgeons to stain the internal limiting membrane (ILM) and epiretinal membrane in macular surgery [1]. 

Methylene blue, while not a vital ocular dye, is commonly found in operating rooms. It is used in urology to aid in identifying ureteral fistulas during procedures, assessing the integrity of the urinary tract, and aid in the diagnosis of urethral strictures. The dye is also used during endoscopies to identify areas of dysplasia on the mucosa of the gastrointestinal tract and to aid sentinel lymph node mapping in cancer surgery in surgical oncology [2]. In ophthalmology, methylene blue has been utilized for external applications such as eyelid surgery for skin incision marking [3, 4]. Outside of the operating room, intravenous methylene blue is FDA approved for the treatment of methemoglobinemia.

Methylene blue’s similar appearance to the ocular blue vital dyes such as trypan blue and brilliant blue may result in its accidental intraocular use in rare cases. In our case, the methylene blue was mistaken for brilliant blue and injected into the vitreous chamber. There was evidence of severe retinal toxicity as shown by the inner retinal layer reflectivity with no evidence of damage to the anterior segment structures. Given the normal appearing fluorescein angiogram, we suspect the retinal toxicity noted was most likely at a cellular level rather than an inflammatory mediated occlusive vasculopathy. In keeping with the reported cytotoxicity of methylene blue which is attributed to its capacity to generate reactive oxygen species (ROS) and inhibit the mitochondrial complex (cytochrome c oxidase), thereby disrupting the electron transport chain and impairing oxidative phosphorylation [2]. This dual mechanism precipitates mitochondrial dysfunction and oxidative stress, ultimately culminating in apoptotic cell death [5]. Furthermore, the drug’s low molecular weight and lipophilicity facilitate rapid intracellular penetration, likely contributing to its toxicity of ocular tissues [6]. Another possible hypothesis underlying methylene blue’s retinal toxicity may be related to its vasoconstrictive properties [7]. The inhibition of guanylate cyclase by methylene blue reduces nitric oxide (NO) production leading to vasoconstriction. Due to these properties, methylene blue has been used in patients with vasodilatory shock [7]. Given that NO plays a pivotal role in maintaining retinal arterial tone, we alternatively hypothesize that the administration of methylene blue caused a transient vasoconstriction event resulting in an ischemic event primarily affecting the inner retinal layers. This explanation seems plausible given the initial OCT findings of diffuse inner retinal layer hyperreflectivity and edema as one would encounter in an arterial occlusion while the outer retina layers appeared largely unaffected.

There are also previously reported cases where methylene blue was mistaken for trypan blue. Alabassi et al. reported 3 cases of inadvertent intracameral injection of 1% methylene blue during cataract surgery [8]. All 3 eyes developed severe corneal edema requiring use of prednisolone acetate 1% every 1 h (Q1), with 2 eyes eventually requiring endothelial keratoplasty (EK) for persistent corneal edema. Brouzas et al. reported another case of severe corneal toxicity and iris discoloration following inadvertent intracameral methylene blue administration during cataract surgery [9]. The patient eventually underwent a full thickness corneal transplant 1.5 years after the initial procedure for persistent corneal edema. Histological examination of the removed cornea posterior lamella demonstrated corneal edema and absence of corneal endothelial cells. Timucin et al. reported a similar case of methylene blue-related bullous keratopathy following intracameral injection [10]. 

Methylene blue’s toxicity to the anterior and posterior segment structures is evident. The question remains; how do we mitigate the risk of these errors? Possible strategies include avoiding storage of look-alike vials in the same area and in this case particularly, of similar appearing dyes. Additionally, medications meant for intraocular use should have a clear labeling stating “intraocular use only” in a bold, large font to minimize the risk of error. Finally, we propose a safety time out to be performed in which the label is both read aloud by the staff obtaining the vital dye and then shown to the operating surgeon prior to use.

In conclusion, while blue vital dyes remain an integral part of ophthalmic surgery, better safety checks are needed to prevent the accidental mix-up of these dyes with methylene blue.

## Data Availability

No datasets were generated or analysed during the current study.
